# An Octahedral Fibrous Constitutive Model for Heart Valve Mechanics and Function

**Published:** 2026-06-02

**Authors:** Nishan Parvez, Prashant K. Purohit, Wensi Wu

**Affiliations:** a Department of Anesthesiology and Critical Care Medicine, Children’s Hospital of Philadelphia, Philadelphia, 19104, Pennsylvania, USA; b Department of Mechanical Engineering and Applied Mechanics, University of Pennsylvania, Philadelphia, 19104, Pennsylvania, USA; c Department of Pediatric Cardiology, Children’s Hospital of Philadelphia, Philadelphia, 19104, Pennsylvania, USA; d Cardiovascular Institute, Children’s Hospital of Philadelphia, Philadelphia, 19104, Pennsylvania, USA

**Keywords:** Hyperelasticity, Anisotropy, Constitutive Model, Heart Valve

## Abstract

Fibrous soft tissues derive their nonlinear mechanical response from networks of extracellular matrix fibers, whose organization gives rise to strain stiffening, the reverse Poynting effect, and anisotropic mechanical behavior. Motivated by these coupled features, we develop an anisotropic hyperelastic model for fibrous biological tissues that accounts for the contribution of the fiber network under both tensile and compressive deformation. We calibrate the model to experimental data for mitral valve leaflets using an inverse finite element approach that is coupled to automatic differentiation to facilitate efficient parameter calibration. Using the calibrated model, we investigate how anisotropy and fiber reorientation affect valve deformation under physiological loading. The results show that greater leaflet compliance in the radial direction yields proper valve closure, whereas localized fiber reorientation leads to stress concentrations that may promote progressive functional degradation. Fiber reorientation that makes the circumferential direction more compliant than the radial direction compromises valve closure and leads to mitral regurgitation. Chordal softening further amplifies the severity of this regurgitant response. These findings suggest that alterations in fiber architecture, especially when accompanied by chordal degradation, can contribute to the onset and progression of mitral valve incompetence.

## 1. Introduction

Soft fibrous materials functioning as load-bearing components of biological tissues and scaffolds of organic matter are ubiquitous in nature [1, 2, 3]. The mechanical behavior of these materials arises from the collective deformation of fibers connected in disordered or partially aligned networks. Under load, the fibers in such networks can rotate, stretch, bend, and reorganize in a non-affine manner, giving rise to nonlinear stiffening and non-classical shear-induced normal stress effects such as the reverse Poynting effect [4, 5, 6, 7]. The same network architecture also determines the degree of anisotropy, which depends on tissue type, physiological function, and the conditions under which the fiber network forms. Heart valve leaflets provide a representative example: their collagen fibers are preferentially arranged along the circumferential and radial directions, leading to markedly different material responses along these axes [8, 9, 10]. Similar structure-function relations arise in blood vessels, tendons, ligaments, and other fibrous tissues, where fiber orientation and dispersion govern the macroscopic response [11, 12, 13, 2]. These structural features are critical to tissue function and motivate constitutive models that can capture anisotropic fibrous mechanics under physiological loading.

Theoretical modeling of fibrous materials has historically followed two broad approaches: discrete material models and continuum constitutive models. Discrete fiber network models explicitly represent fibers and their interactions through crosslinks, contacts, or filament-level mechanics. Finite element and related methods have been used to study networks of athermal fibers such as collagen, revealing how network connectivity and fiber bending control non-affine deformation and strain stiffening [14, 15, 16, 17, 18]. However, the computational cost of these models limits their direct application to small systems and idealized loading conditions. As such, continuum constitutive models provide a more practical route for finite element analysis (FEA) of complex geometries and boundary value problems [15, 19, 20, 21, 22, 23]. Although these models represent fiber-level kinematics through effective constitutive assumptions rather than by explicitly resolving deformation of the microscopic network, they remain standard in FEA of fibrous tissue behavior. Consequently, their predictive capability depends strongly on how fiber-network contributions are incorporated into the constitutive law.

A central assumption in many existing constitutive models concerns the mechanical role of fibers under compression. The well-established Holzapfel-Gasser-Ogden (HGO) model, for example, captures anisotropic behavior through a strain-energy function that combines an isotropic ground matrix with distributed fiber contributions [24, 11, 25, 26]. In most HGO-type formulations, the fibers sustain only tensile loads, an assumption usually justified by fiber buckling or negligible compressive resistance. Similar ideas appear in Fung-type models and in isotropic or transversely isotropic formulations developed for heart valve tissues [22, 27, 28]. Although this treatment is effective in many applications, it leaves important network mechanisms unresolved. Fibers may not simply buckle under compression; they may also bend, rotate, and reorganize because of their finite bending stiffness and orientation relative to the loading direction. Discrete network studies show that deformation can remain bending dominated up to moderately large strains [17, 18, 29]. Similarly, fluid efflux during tensile loading can lead to volume reduction in soft tissues, thus incompressibility assumption in various hyperelastic model may be inadequate or fail to capture the actual material behavior [30]. These observations point to the need for a general constitutive model that accounts for fiber-network contributions under both tensile and compressive deformation while representing anisotropic fiber orientation and nonlinear stiffening.

To address this need, we developed an anisotropic hyperelastic constitutive model, termed the octahedral fibrous model, for soft fibrous materials. The model uses fourteen fiber directions to represent a fibrous material with orthotropic symmetry, with an isotropic version appearing in [31]. These fibers are embedded in an isotropic ground matrix that represents softer non-fibrous constituents and provides additional resistance to compressive and bulk deformation. The fiber energy is smooth and allows fiber-network contributions under both tensile and compressive deformation. This construction provides a unified framework for modeling anisotropic stiffening and direction-dependent compliance in soft fibrous materials. As a case study, we apply the proposed model to mitral valve deformation under physiological pressure loading. The material parameters are calibrated to biaxial experimental data from porcine mitral valve tissue, and the calibrated model is used to examine how anisotropy, global and localized fiber reorientation, and chordae tendineae degradation influence valve mechanics and closure quality. We find that an isotropic approximation of tissue constitutive behavior does not lead to noticeable differences in global deformation, but fiber reorientation can influence valve closure. Specifically, reorientation that increases circumferential compliance to a greater extent than radial compliance compromises coaptation and promotes mitral regurgitation. Chordal degradation further exacerbates this response. These results suggest that altered leaflet microstructure may introduce a mechanical risk that impairs valve closure.

## 2. Material Model and Methods

### 2.1. Strain Energy Density Function for Octahedral Fibrous Model

**Figure 1:**
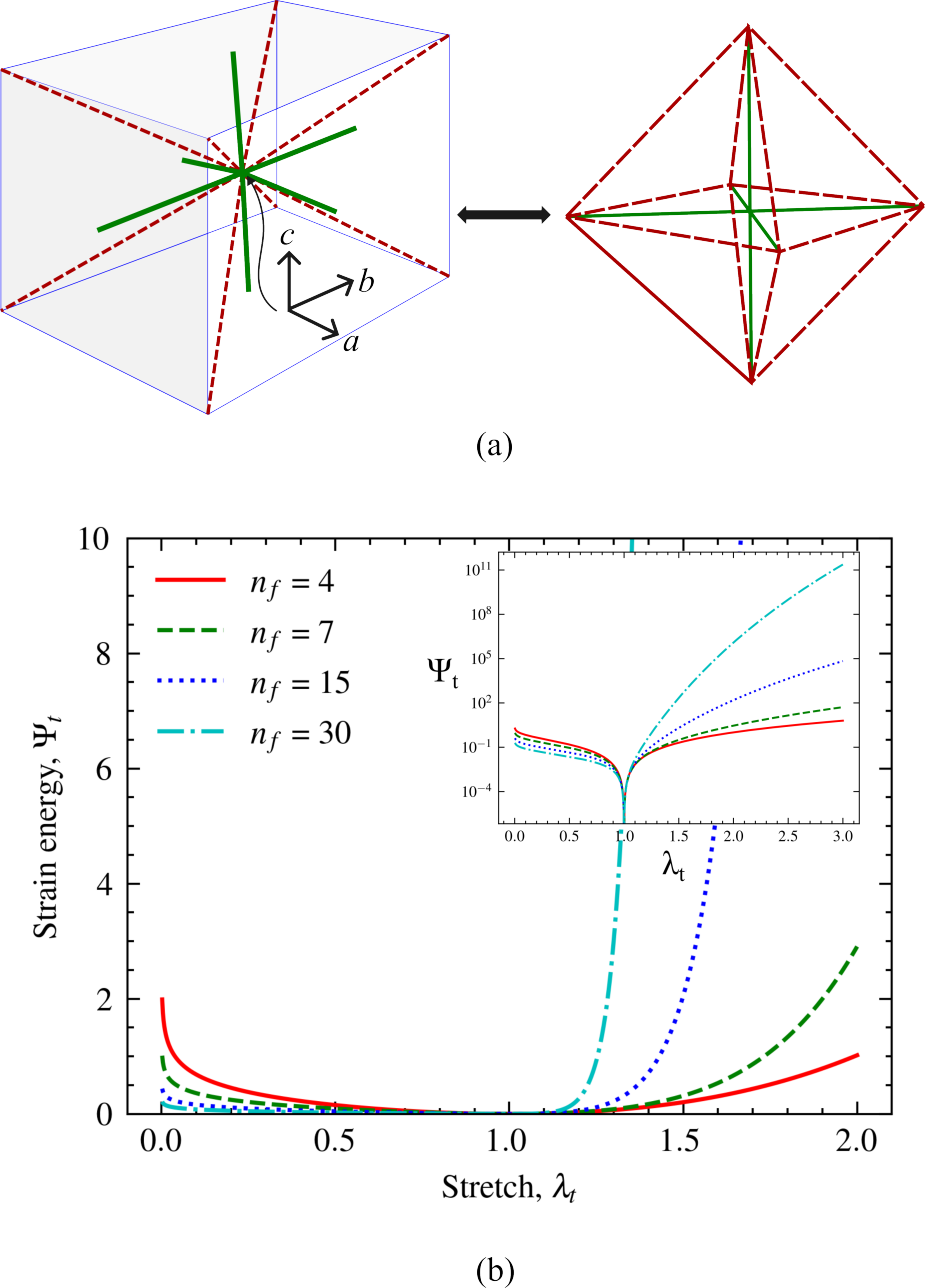
(a) Schematic of the unit cell describing fiber placement for the proposed material model. The solid green and dashed red lines indicate (i) centerto-face and (ii) center-to-corner fibers, respectively, with origin at the center. The overall fiber arrangement in the cuboid is equivalent to an octahedron with fibers along the edges and internal diagonals. (b) Strain energy of a single fiber with unit length and stiffness as a function of stretch. A more comprehensive view is shown as an inset.

The mechanical behavior of stochastic network materials emanates from the mechanical properties of the fibers and their interactions. In defining a strain energy density function from a continuum mechanics viewpoint, we coarse-grain the micromechanical details of an otherwise complex material system while capturing certain key features. To this end, we envision that a representative volume element of the material is equivalent to a network of 14 ‘fibers’ organized along the centerlines and diagonals of a cuboid shown in Figure 1a. An equivalent representation of the fiber arrangement is placing fibers along the edges and inner diagonals of an octahedron, as shown in the same figure. This particular fiber placement is a generalization of a model proposed in [17] where it was postulated that exponential stiffening in fibrous materials is a consequence of geometric non-linearities associated with deformation of fibers in an equivalent unit cell. In contrast to a discrete collagen fiber with bending rigidity, it is, however, convenient to imagine the ‘fibers’ in our model as a fiber bundle structured as a helical spring that is thematically similar to stress-path/force chain concepts explored in literature [17, 32, 33]. Other works also utilized a similar unit cell with differences in the energy function and nature of the fiber/chain, for example, in [34, 35, 36, 37, 38]. Our advancement lies in the inclusion of tension-compression behavior of the fibers, anisotropic nature of the overall material in a single framework, and its feasibility in modeling soft fibrous materials as presented in later sections of this work.

We consider two sets of such fibers: (i) center-to-face fibers that connect the center of the cell to the centers of the faces, and (ii) center-to-corner fibers that connect the center to the corners in the cuboid view. With a, b and c denoting the half-lengths of the cuboid unit cell, the fibers are oriented along the following directions in the local coordinate system:

(1) $$\vec{a}=(a,\;0,\;0)\;\vec{b}=(0,\;b,\;0)\;\vec{c}=(0,\;0,\;c)\;\vec{p_{1}}=\vec{a}+\vec{b}+\vec{c}\;\vec{p_{2}}=\vec{a}+\vec{b}-\vec{c}\;\vec{p_{3}}=\vec{a}-\vec{b}+\vec{c}\;\vec{p_{4}}=\vec{a}-\vec{b}-\vec{c}$$

Here, $\vec{a}$, $\vec{b}$, $\vec{c}$ are the directions of the center-to-face fibers that double as the direction of the local axes. Vectors ${\vec{p}}_{1\ldots 4}$ represent the center-to-corner fibers. Since we consider the fibers to emanate from the center of the cell, the fiber orientations are defined by the vectors above and their negatives, totaling 14 fibers in the unit cell. We define the strain energy of a fiber as a function of its stretch, $\lambda_{t}$. For an applied deformation gradient F, this stretch is calculated as:

(2) $$\lambda_{t}=\frac{\sqrt{{\vec{v}}^{T}F^{T}F\vec{v}}}{\left\|\vec{v}\right\|}=\frac{\sqrt{{\vec{v}}^{T}C\vec{v}}}{\left\|\vec{v}\right\|}$$

Here, $C=F^{T}F$ is the right Cauchy-Green deformation tensor. Vector $\vec{v}$ takes the appropriate value for the target fiber from Eq. 1. The strain energy is then defined as follows for the center-to-face fibers:

(3) $${\text{Ψ}}_{t}=\frac{E_{f}L_{f}}{n_{f}\;-\;1}\left(\frac{1}{n_{f}}\lambda_{t}^{n_{f}}-\log\lambda_{t}\right)+k$$

Here, $E_{f}$ is the stiffness of the fiber, $n_{f}$ is a non-linearity parameter that controls the degree of stiffening, $L_{f}$ is the initial length of the vectors denoting a fiber (e.g., a, b or c). In the definition, k is an arbitrary constant that one may consider to be zero without any loss of generality. The energy for center-to-corner fibers is expressed following the same template with $E_{c}$ and $n_{c}$ denoting the stiffness and non-linearity parameter, respectively. The initial fiber lengths are the magnitude of vectors ${\vec{p}}_{1\ldots 4}$. This particular energy function formulation is due to works such as [39].

From observation, ${\text{Ψ}}_{t}\to \infty$ as $\lambda_{t}\to \infty$ due to the power term. The $-\log\lambda_{t}$ term ensures that ${\text{Ψ}}_{t}\to \infty$ as the fiber is compressed (i.e., $\lambda_{t}\to 0$) with minima at $\lambda_{t}=1$. Therefore, the fiber model is designed to sustain both tension and compression with a smooth transition. This contrasts with many popular hyperelastic models that engage the fibers only under tension. The strain energy function is visualized in Figure 1b for several values of the exponents. A detailed discussion of the contribution of the fiber to the overall stress and construction of the tangent stiffness is presented in Appendix A.

In addition to stiff collagen fibers, tissue also contain elastin fibrils and other biopolymers. We model their contribution as an isotropic ground matrix using a compressible Neo-Hookean strain energy density defined below [40]:

(4) $$\psi_{nh}=\frac{\mu_{nh}}{2}\left(I_{1}-3\right)-\mu_{nh}\log J+\frac{\lambda_{nh}}{2}{\left(\log J\right)}^{2},$$

where $\mu_{nh}$ and $\lambda_{nh}$ are the Lamé parameters, $I_{1}$ is the first invariant of the right Cauchy-Green deformation tensor, and J is the determinant of the deformation gradient. This introduces two additional material parameters, small strain stiffness, $E_{n}$ and Poisson’s ratio, v, through $\mu_{nh}=\frac{E_{n}}{2(1+v)}$ and $\lambda_{nh}=\frac{E_{n}v}{\left(1+v)(1-2v\right)}$. The overall strain energy density function for our proposed material model is then given by:

(5) $$\psi =\psi_{\text{aniso}}+\psi_{\text{iso}}=\frac{1}{V_{o}}\left(\sum_{i=1}^{6}\Psi_{t,\text{face}}^{\left(i\right)}+\sum_{j=1}^{8}\Psi_{t,\text{corner}}^{\left(j\right)}\right)+\psi_{nh}$$

Here, $V_{o}=abc$ is the volume of the unit cell in the reference configuration. In summary, the proposed material model is a unit cell composed of non-linear spring-like fibers embedded in a Neo-Hookean medium in a solid-mixture fashion. The choice of Neo-Hookean as the ground matrix is motivated by the convenience of a simpler formulation and a minimum number of additional parameters. Due to known limitations of the Neo-Hookean model under very large deformations, it may be desirable to use alternative models, such as the generalized Mooney-Rivlin model, as the ground matrix [40].

### 2.2. Material Parameter Calibration

Parameter calibration for nonlinear anisotropic constitutive models is challenging because common experimental studies, such as uniaxial and biaxial tests, often do not admit closed-form stress-strain solutions. This difficulty is compounded by the high-dimensional parameter spaces associated with anisotropic formulations. For instance, the proposed material model contains seven independent parameters for the anisotropic part of the model categorized into three sets: (a,b,c), $\left(E_{f},E_{c}\right)$, and $\left(n_{f},n_{c}\right)$. The isotropic ground matrix introduces two additional parameters, $E_{n}$ and v. Thus, the material model contains a total of 9 parameters. We require $E_{f}$, $E_{c}$, $E_{n}$, a, b, c>0 and −1<v<0.5. A stability criterion discussed in Appendix C requires $n_{f}$, $n_{c}>2$. The large number of parameters allows for extensive flexibility in modeling various behaviors of fibrous materials. However, it also complicates the calibration process.

In the literature, inverse FEA is commonly adopted for material parameter calibration, where material parameters are identified by coupling FEA with an optimization algorithm [41]. However, derivative-free methods such as Nelder-Mead become computationally expensive when each objective-function evaluation requires a full finite element solve. This limitation is particularly pronounced for the proposed octahedral constitutive model, which involves a nine-dimensional parameter space. To mitigate this computational cost, we streamline parameter calibration by coupling FEBio finite element simulations with an automatic differentiation optimization framework implemented in JAX [42, 43]. This procedure follows the general idea of Finite Element Model Updating (FEMU) [41, 44].

The optimization workflow proceeds as follows. The octahedral strain-energy density function is implemented in both FEBio and JAX. FEBio is used to solve the boundary value problem and extract the deformation gradients, whereas JAX evaluates the strain-energy function, computes the stress response, and differentiates the loss with respect to the material parameters. To minimize computational cost, the biaxial test is modeled in FEBio using a single brick element. The resulting deformation gradients are passed to JAX, where the predicted stresses are compared with the experimental data using an MSE loss augmented by penalty terms for material constraints and symmetry conditions. The Adam optimizer then updates the material parameters using the gradients of this loss. Because the deformation field may change as the material parameters evolve, the FEBio solution is recomputed iteratively during optimization. The workflow is discussed in Appendix B in greater detail. We will share our implementation in https://github.com/wu-lab-crisp/octahedral-fibrous-material-model once the paper is accepted, to enable other researchers to easily adapt this approach.

## 3. Results

### 3.1. Modeling General Behavior of Fibrous Materials

#### 3.1.1. Parameter Calibration to Experimental Data

**Figure 2:**
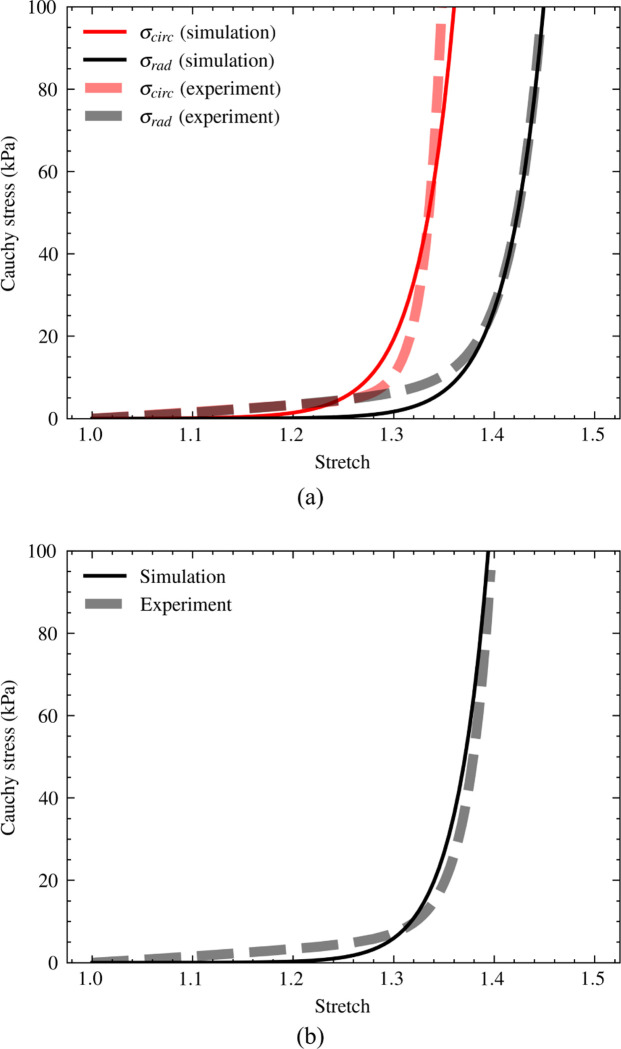
Stress-stretch of a mitral valve tissue sample under equi-biaxial load predicted by the fitted material model with (a) transverse isotropic and (b) isotropic assumption. The experimental and fitted results are shown using dashed and solid lines, respectively. Fitted material parameters for (a): *b* = 13.59, *a* = *c* = 2.77, *E*_*f*_ = 210.05, *E*_*c*_ = 207.58, *n*_*f*_ = 29.17, *n*_*c*_ = 29.44, *E*_*n*_ = 114.95, *ν* = 0.23, and for (b): *a* = *b* = *c* = 6.26, *E*_*f*_ = 204.89, *E*_*c*_ = 217.79, *n*_*f*_ = 38.36, *n*_*c*_ = 37.69, *E*_*n*_ = 169.97, *ν* = 0.30.

Using the optimization framework described in Sec 2.2, we calibrate our material model to the response of a tissue sample from the porcine mitral valve under a biaxial test setup from Ref. [10]. We constrained the material parameters during optimization such that the overall model is transversely isotropic. The material parameter optimization was performed on a MacBook (M4 Pro, 48 GB RAM). The overall procedure required only a few minutes of wall time (about a thousand iterations) to reach an acceptable solution from a random starting point. The calibrated material parameters are b=13.59, a=c=2.77, $E_{f}=210.05$, $E_{c}=207.58$, $n_{f}=29.17$, $n_{c}=29.44$, $E_{n}=114.95$, v=0.23. We also fit the material model to an isotropic approximation of the same data (obtained through averaging of the radial and circumferential stress-stretch responses) with constraints a=b=c. We obtain the following values of material parameters in this case: a=b=c=6.26, $E_{f}=204.89$, $E_{c}=217.79$, $n_{f}=38.36$, $n_{c}=37.69$, $E_{n}=169.97$, v=0.30. The experimental data and predictions based on the calibrated material parameters are shown in Figure 2a and 2b for the transversely isotropic and isotropic assumptions. In both cases, the fitted material model captures the experimental data well. The coefficient of determination indicated strong model-data agreement, with $R^{2}=0.91$ and 0.97 in the circumferential and radial directions, respectively. For isotropic approximation, $R^{2}\approx 0.92$.

#### 3.1.2. Modeling Stiffening and Volumetric Behavior

In modeling stochastic fibrous networks with a hyperelastic material model, we aim to capture properties that make them unique. Here we focus on stiffening and volume reduction under tension [5, 18, 45, 30]. Due to the mathematical complexity of the model, the discussions are limited to results from numerical experiments. We consider the case of the reverse Poynting effect in the next section.

**Figure 3:**
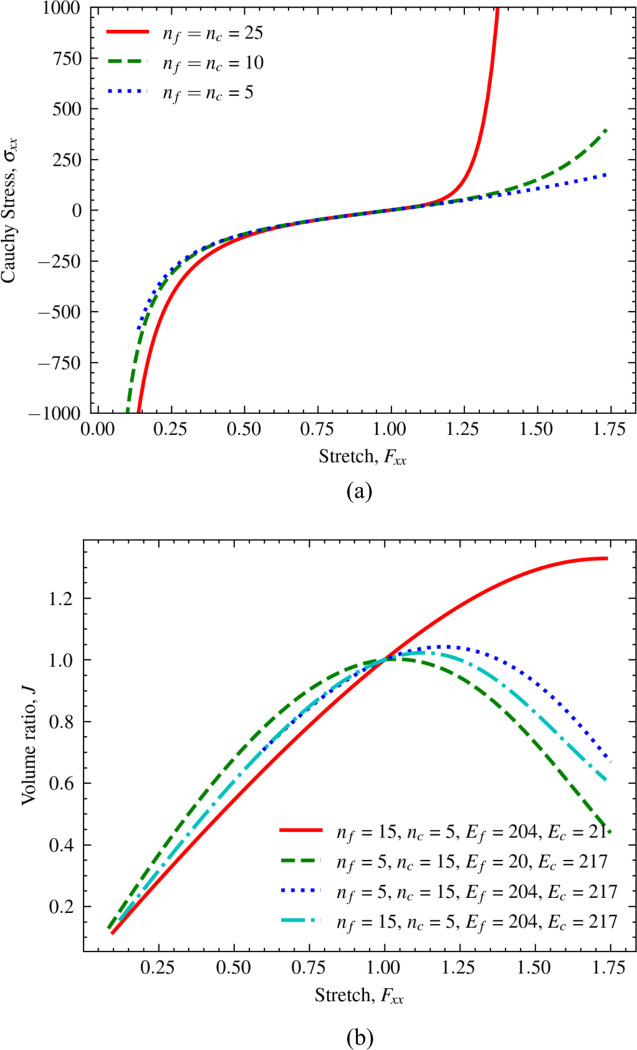
(a) Stress-stretch relation for the material model and (b) volume ratio under uniaxial tension/compression. Values for material parameters other than those shown in the legend are set to those reported for Figure 2b (isotropic assumption).

In the proposed octahedral model, the exponents, $n_{f}$ and $n_{c}$, primarily control the nature of the stiffening and the extent of the toe-regime in the characteristic J-shaped curve. Examples of this are shown in Figure 3a, where increasing the value of $n_{f}\left(=n_{c}\right)$ leads to early stiffening of the material under uniaxial deformation. Other material parameters are kept equal to the fitted material parameters for isotropic approximation shown in Figure 2b.

Fibrous materials, especially in the absence of stiff or incompressible background media, experience strong Poisson’s contraction under uniaxial tension that can lead to J=det(F)<1 [18, 46, 30]. The octahedral model can accommodate J<1, J>1 and their combination through deliberate choices of material parameters. Due to a lack of an analytical solution describing the relation between $\lambda_{1}$ to $\lambda_{2}$, $\lambda_{3}$ under uniaxial tension, we cannot directly identify which material parameters, or combinations thereof, govern this behavior. Numerical experiments for a cube under uniaxial tension/compression suggest $n_{f}>n_{c}$ with $E_{f}>E_{c}$ results in J>1 up to a very large stretch. In contrast, $n_{f}<n_{c}$ with $E_{f}<E_{c}$ leads to J<1 under tension in absence of a stiff ground matrix. The strain at which the behavior shifts can be controlled through a combination of these parameters. Results summarizing this behavior are shown in Figure 3b.

The anisotropy in the proposed model is primarily controlled by the relative magnitude of parameters a, b and c with a clear geometric interpretation. The relative magnitude of these parameters, in essence, controls how aligned the center-to-corner fibers are to an external load in a principal direction. This interpretation parallels the kinematics of the discrete fibers in stochastic network material, where anisotropy originates from the preferential alignment of fibers.

#### 3.1.3. Poynting Effect

Biological soft tissues are known to exhibit a strong reverse Poynting effect in simple shear [4, 7, 47, 48]. This is a phenomenon where the material tends to contract in the transverse direction when subjected to simple shear deformation. This is a consequence of the deformation of the fibers non-parallel to the shear direction. Due to the specific arrangement of the fibers in the unit cell, our material model clearly captures this behavior. To make it concrete, we consider a deformation gradient of the form:

(6) $$F=\left[\begin{array}{lll} 1 & \gamma & 0\\ 0 & 1 & 0\\ 0 & 0 & 1 \end{array}\right],$$

where γ is the shear strain. Under this deformation, the center-to-face fibers that are perpendicular to the shear direction stretch along and perpendicular to the shear direction. The center-to-corner fibers, on the other hand, form two groups and deform in a complex manner. For instance, for the above deformation gradient and fiber orientation described in Eq. 1, the stretches in the center-to-face fibers are:

(7) $$\lambda_{a}=1\;\lambda_{b}=\sqrt{1+\gamma^{2}}\geq 1\;\lambda_{c}=1$$

For the center-to-corner fibers (with $r^{2}=a^{2}+b^{2}+c^{2}$):

(8) $$\lambda_{p1}=\sqrt{\frac{a^{2}\;+\;2\gamma ab\;+\;\left(1\;+\;\gamma^{2}\right)b^{2}\;+\;c^{2}}{a^{2}\;+\;b^{2}\;+\;c^{2}}}\;\;=\sqrt{1\;+\;\frac{b^{2}\gamma^{2}\;+\;2\gamma ab}{r^{2}}}\;\lambda_{p2}=\sqrt{1\;+\;\frac{b^{2}\gamma^{2}\;+\;2\gamma ab}{r^{2}}}\;\lambda_{p3}=\sqrt{1\;+\;\frac{b^{2}\gamma^{2}\;-\;2\gamma ab}{r^{2}}}\;\lambda_{p4}=\sqrt{1\;+\;\frac{b^{2}\gamma^{2}\;-\;2\gamma ab}{r^{2}}}$$

The above equations lead to the following transverse PK-2 stress component to $\psi_{\text{aniso}}$ :

(9) $$\begin{array}{l} S_{22}\;\sim \;\frac{E_{f}}{\left(n_{f}\;-\;1\right)}(\lambda_{b}^{n_{f}-2}-\frac{1}{\lambda_{b}^{2}})\\ \;+\;\frac{b^{2}E_{c}}{r^{2}\left(n_{c}\;-\;1\right)}\left[\lambda_{p1}^{n_{c}-2}+\lambda_{p2}^{n_{c}-2}+\lambda_{p3}^{n_{c}-2}+\lambda_{p4}^{n_{c}-2}\right.\\ \left.\;-\;\left(\frac{1}{\lambda_{p1}^{2}}+\frac{1}{\lambda_{p2}^{2}}+\frac{1}{\lambda_{p3}^{2}}+\frac{1}{\lambda_{p4}^{2}}\right)\right] \end{array}$$

**Figure 4:**
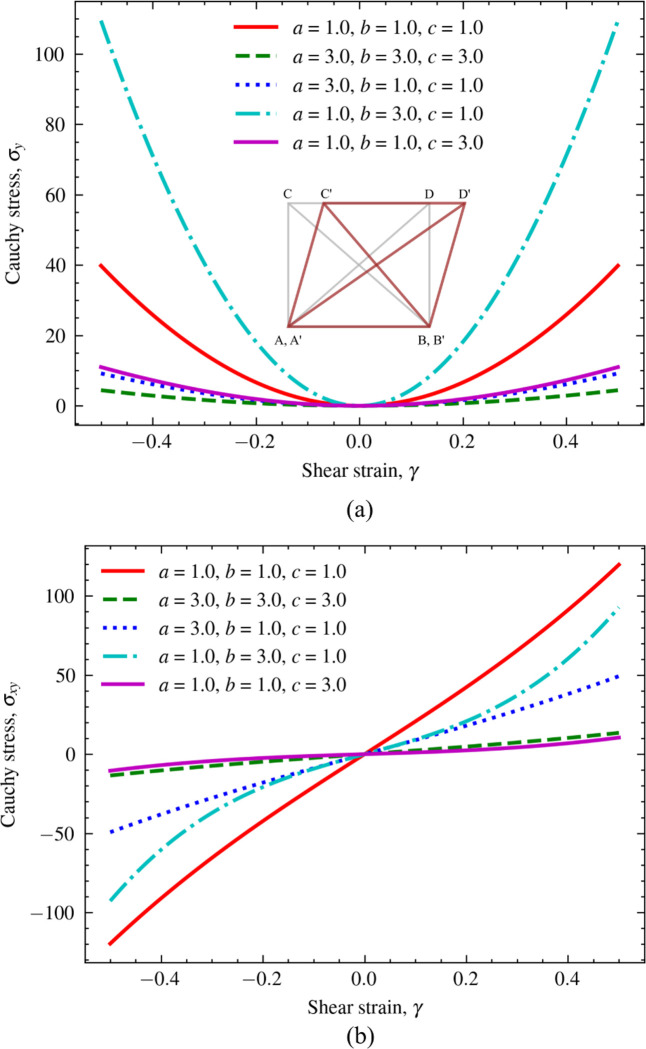
(a) Numerical results showing reverse Poynting effect under simple shear in the proposed material model for various choices of *a*, *b* and *c*. A schematic of the mechanism driving this phenomenon is shown as an inset. (b) General stress-strain response of the model under simple shear.

From Eq. 7, $\lambda_{b}\geq 1$. The stretches $\lambda_{p1\ldots 4}$ are offset from $1+b^{2}\gamma^{2}/r^{2}\geq 1$ by ±2γab. Consequently, ±2γab does not contribute to the sum in Eq. 9. Similarly, the isotropic Neo-Hookean background also does not contribute to the Poynting effect [48]. Thus, the material model produces $S_{22}\;\sim \;\sigma_{22}\;>\;0$ (*reverse Poynting effect*). Numerical results of this are shown in Figure 4a for several combinations of material parameters a, b and c. The other material parameters are set to $E_{f}=E_{c}=100$, $n_{f}=n_{c}=4$, $E_{n}=1$ and v=0.3. The mechanism controlling this behavior is shown schematically as an inset in the figure with help of a simplified unit cell. In the figure, fiber AC and BD are stretched to $A^{\prime }C^{\prime }$ and $B^{\prime }D^{\prime }$ due to the applied shear with $A=A^{\prime }$ and $B=B^{\prime }$. Their contribution is captured by $\lambda_{b}$ in Eq. 9. The diagonal fiber AD stretches to $A^{\prime }D^{\prime }$ whereas BC is compressed to $B^{\prime }C^{\prime }$, which reflects the discussion regarding $\lambda_{p1\ldots 4}$. Note, this conclusion does not apply when the unit cell is reoriented. In such cases, all center-to-face and center-to-corner fibers may contribute to $S_{22}$, and the reorientation and relative magnitude of the parameters will determine its sign.

The general stress-strain behavior of this material under shear is shown in Figure 4b with material parameters kept the same as in Figure 4a. The overall response is a combination of various material parameters that are not explored here for brevity. However, it is clear that the model can capture non-linearity in shear without difficulty.

#### 3.1.4. Compression, Stability, and Contribution of Neo-Hookean

The requirement of objectivity for the proposed model is satisfied by construction. It is also easy to show that the anisotropic strain energy density is a convex function of C for $n_{f}$, $n_{c}\geq 2$, and so the tangent stiffness is positive definite (see Appendix C). The functional form, however, does not allow us to make any conclusive remark on polyconvexity.

In practice, we do not encounter any convergence issues up to a very large strain for simple boundary value problems such as uniaxial and biaxial tension with or without $\psi_{iso}$ The same is true up to a moderately large degree of compression. Thus, the stability of the proposed model does not necessarily depend on the existence of the Neo-Hookean matrix. However, the matrix plays a critical role in our ability to model tissue materials and perform complex numerical simulations, particularly those under compression.

The response of the material model under compression is controlled by the competition between the power and logarithmic term in the strain energy function defined in Eq. 3 (visualized in Figure 1b). For a single fiber, the relative energetic penalty for extension is significantly larger than in compression until near full compaction. For the unit cell under uniaxial tension, it is thus energetically favorable for the fibers other than those in the loading direction to contract, leading to Poisson’s contraction. Under uniaxial compression, on the other hand, the propensity of the fibers other than those in the loading direction to extend is determined by the relative energy penalty of the log $\lambda_{t}$ term (active for the fibers in the loading direction) and $\lambda_{t}^{n}$ term (active for remaining fibers). For large $n_{f}$, $n_{c}$, it may be energetically favorable to deform such that $\lambda_{t}\to 1$ for the fibers in tension (transverse fibers). Under such a scenario, one should expect $J∼F_{xx}$, where $F_{xx}$ is the applied compressive stretch. Anisotropy (e.g., a>b=c) with large $n_{f}/n_{c}$ does allow us to control this behavior somewhat with J>1 in compression up to some strain. However, J<1 in compression remains the predominant behavior for the material model.

**Figure 5:**
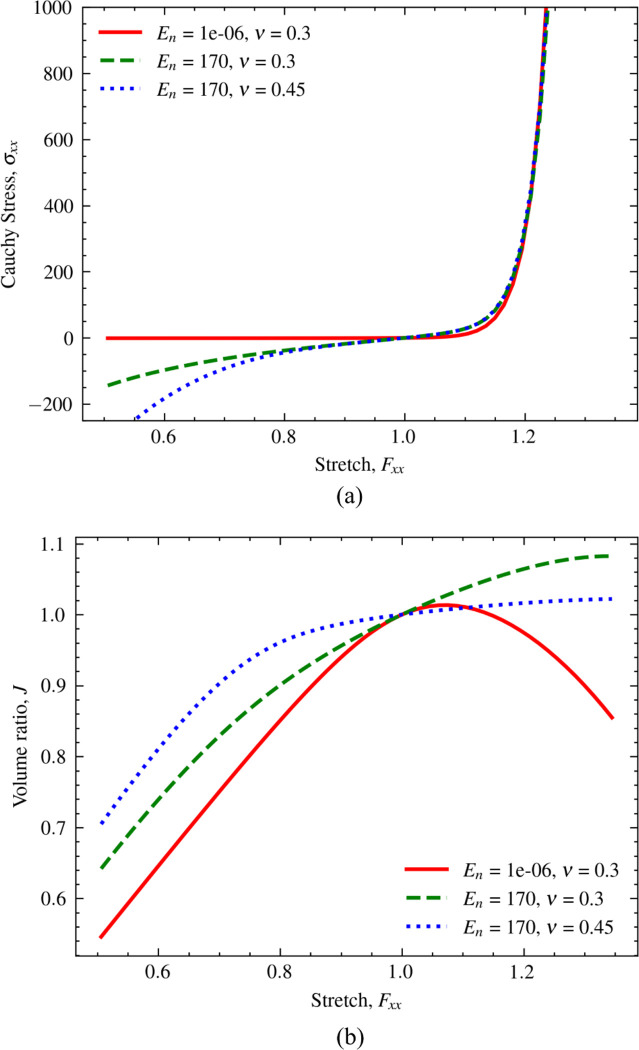
Effect of Neo-Hookean contribution (material parameter *E*_*n*_ and *ν*) on the (a) stress-stretch and (b) volumetric ratio under uniaxial tension and compression. Values for material parameters other than those shown in the legend are set to those reported for Figure 2b (isotropic assumption).

As is clear from Figure 1b, the anisotropic part of the material model remains very compliant until very large compression for large $n_{f}$, $n_{c}$. Computational studies of network materials without a background medium show this is indeed expected [14]. However, it leads to numerical convergence issues, for instance, in complex boundary value problems such as heart valve deformation presented later. In such cases, including Neo-Hookean matrix significantly helps with the numerical stability by improving the resistance to compressive load. This is evidenced by results presented in Figure 5a. It is clear from this result that the contribution of Neo-Hookean matrix to the overall stress production under tension is negligible, especially when $n_{f}$, $n_{c}$ in $\psi_{\text{aniso}}$ are large or Neo-Hookean parameters take relatively smaller values. However, it strongly contributes to the behavior of the material under compression as $E_{n}$ and/or v increases. It also guards against possible floppy modes or instabilities in the fiber system [49, 15]. Finally, it provides a direct pathway to manipulate the compressibility of the material as shown in Figure 5b. However, since the Neo-Hookean formulation penalizes compressibility through log terms, the preceding discussion remains valid and under very large compression, J→0 even with v→0.5 due to competition between the power and logarithmic terms. This can be mitigated by adjusting other material parameters such as $E_{f}$, $E_{c}$ if the flexibility exists. Alternatively, one may utilize other energy functions that can penalize the volumetric compression and are potentially more suitable as a ground matrix model for problems involving very large tension/compression, e.g., in [50].

### 3.2. Case Study: Mitral Valve Deformation

Numerous experimental results conclusively show that mitral valve tissue exhibits anisotropic behavior with orthogonal directions [9, 10]. Here, we demonstrate how our model can be used through numerical simulation of a mitral heart valve model with transversely isotropic material properties. We also investigate the role of anisotropy and fiber orientation on the mechanical behavior of the mitral valve. Finally, we identify risk factors that may emerge due to fiber reorientation and other reasons.

#### 3.2.1. FEA model and fiber orientation setup

**Figure 6:**
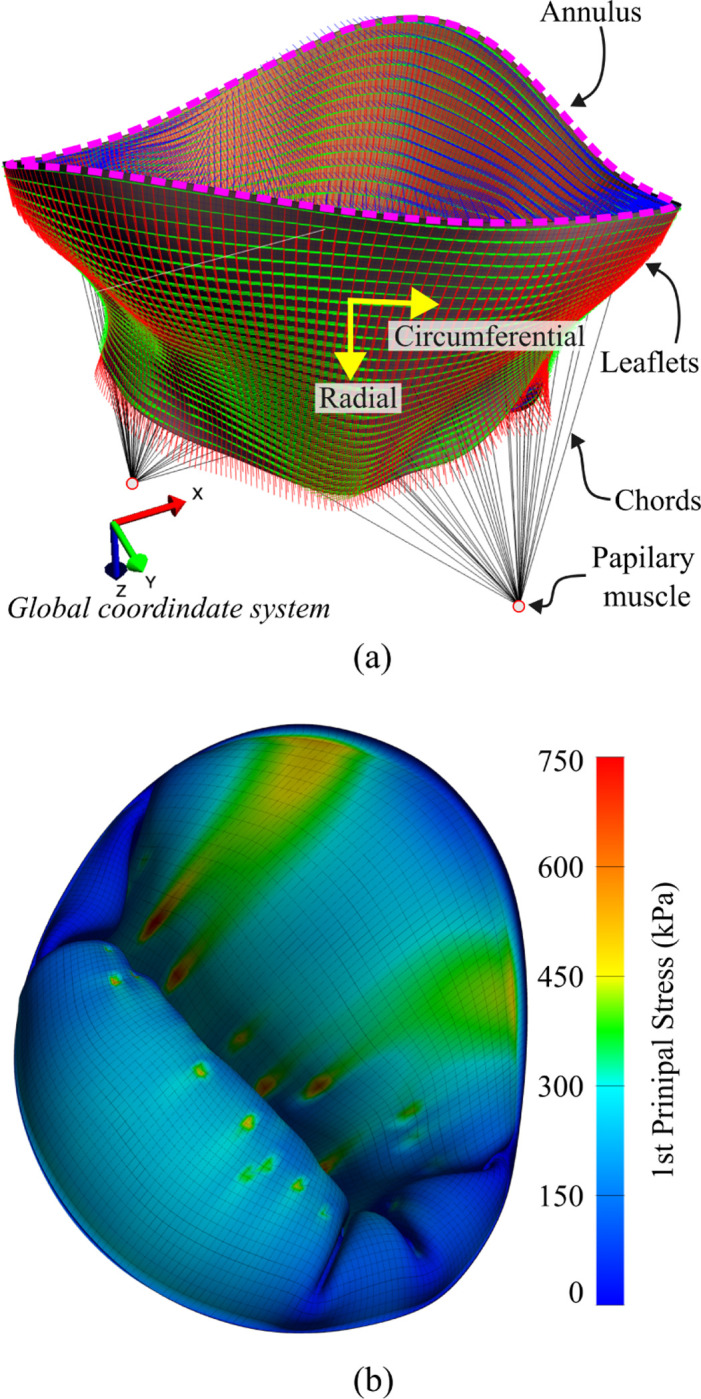
(a) Finite element model of the mitral valve. The fiber orientations (local/material axes) are from the solution of a Poisson equation. The local y-axis (green) is aligned with the circumferential direction, and the x-axis (red) is aligned with the radial direction. Pressure load is applied on the outer surface of the leaflets. (b) The deformed configuration of the value at maximum applied pressure (10 kPa). The colors show the first principal value of Cauchy stress in kPa.

For this case study, we utilize the FE model of the mitral valve shown in Figure 6a. We consider the leaflet material to be transversely isotropic with fibers oriented along the circumferential and radial directions, as shown in the Figure 6a. The chordae tendinae are modeled with line elements with one end attached to random points on the leaflet, while the other ends are anchored to two fixed positions. We use the proposed material model for the leaflet only.

Several techniques, e.g., in Ref [51], can be used to obtain the fiber orientation that is compatible with the geometry of the valve. Here, we use a simple approach where the local fiber orientations are defined based on the solution of Poisson’s equation implemented in the FEBio FEA package (Fiber Generator Tool). In this paradigm, prescribed scalar boundary values are defined at the annular and free edge of the valve, and the solution of Poisson’s equation is used to define the local orthonormal basis vectors. The fiber orientation thus generated is visually comparable to those reported based on experimental techniques [10, 9]. The fiber orientations can be perturbed by modifying the constraints or through random perturbation of the generated vectors.

A physiologically relevant pressure of 10 kPa is applied to the outer surface of the leaflet. A normal displacement is applied to the elements at the annular ring to mimic the deformation of the valve in vivo. We utilize a shell element (QUAD4, thickness equals 0.39 mm) and a fitted material model presented in Figure 2 as the leaflet material. We utilize potential-based contact mechanics in the simulation for the leaflet [52]. The chordae tendinae are modeled using elastic truss elements with an unconstrained Ogden material model defined below [53]:

(10) $$\psi_{\text{Ogden}}=c_{p}{\left(J-1\right)}^{2}+\sum_{k=1}^{N}\frac{c_{k}}{m_{k}}\left(\lambda_{1}^{m_{k}}+\lambda_{2}^{m_{k}}+\lambda_{3}^{m_{k}}-3-m_{k}\log J\right)$$

The choice of this material model is motivated by the large exponents for the leaflet material after calibration, though a simple linear truss also performs well. The chosen value of material parameters for this work is arbitrary with N=1, $c_{p}=10$, $c_{k}=100$, and $m_{k}=15$.

#### 3.2.2. Transverse Isotropy vs Isotropy

We start the discussion by presenting the deformation and valve closure for the transversely isotropic material model. With the calibrated material parameters, the valve fully closes at approximately 6 kPa applied pressure. The deformed configuration of the valve at maximum applied 10 kPa pressure is shown in Figure 6b. The color bar shows the first principal stress (clipped at 750 kPa). The distribution of the first principal stress is exponential-like with a maximum value of 3.5 MPa, a mean of 156 kPa, and a median of 134 kPa. The peak stress is associated with the elements where the chords attach to the leaflet and is likely a consequence of material property mismatch between the chords and the leaflet material, and the simplified model of the chords.

**Figure 7:**
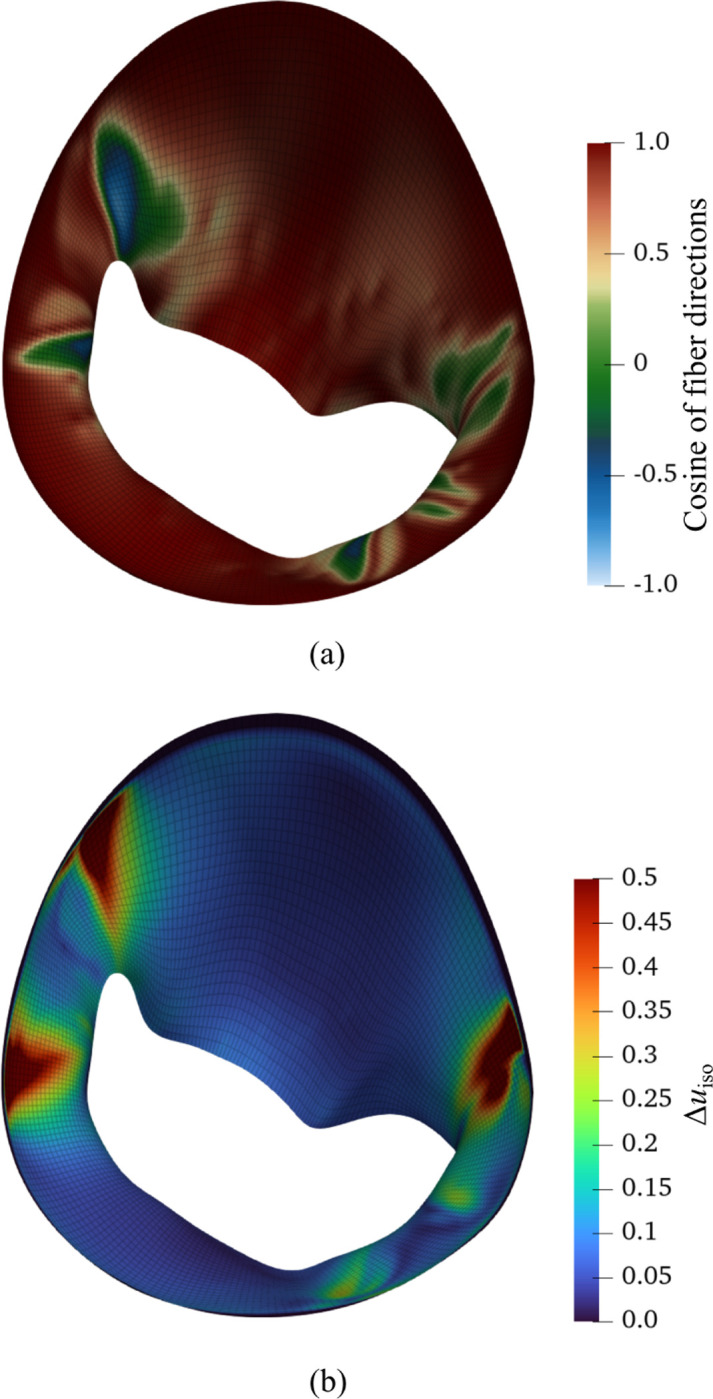
(a) Dot product of the vectors representing the final (at 10 kPa) and initial state of the circumferential fibers, projected to the undeformed configuration (transverse isotropic material). (b) Relative difference in displacement magnitude between the transverse isotropic and isotropic cases at 10 kPa pressure. The color bar is capped at 50% to improve contrast.

The degree of the fiber reorientation upon application of pressure load is quantified in Figure 7a. The figure shows the dot product result of the unit vectors representing the fibers in the circumferential direction in the undeformed and at maximum load state, i.e., ${\vec{v}}_{\text{init}}\cdot {\vec{v}}_{\text{final}}$ with ${\vec{v}}_{\text{final}}=F{\vec{v}}_{\text{init}}$, followed by a normalization. Thus, a value close to one indicates a high degree of alignment between the two states and vice versa. Interestingly, most fibers, especially in the anterior and posterior regions, do not reorient significantly. We observe significant fiber reorientation in the commissure region where complex folding of the leaflet takes place (see Figure 6b). The first principal stresses in these regions have relatively smaller magnitudes than those we find in other parts of the valve. This, along with the overall deformation, suggests that fiber reorientation measured in the lab coordinate system is primarily driven by rigid-body rotations rather than by deformation of unit cells.

We also compare the deformation of the valve with transverse isotropic material to the isotropic approximation from Figure 2b. We do not observe any clinically significant differences, e.g., significant billowing or regurgitation, with the isotropic approximation compared to the arguably more rigorous transversely isotropic material model. The relative differences in the displacement field between these cases are shown in Figure 7b. Here the relative difference have been calculated as $\text{Δ}u_{\text{iso}}=\frac{\left\|{\vec{u}}_{\text{aniso}}-{\vec{u}}_{\text{iso}}\right\|}{\left\|{\vec{u}}_{\text{aniso}}\right\|}$. The differences are mostly concentrated in the commissure region, which follows the observation made for the fiber reorientation. This limited effect of anisotropy on the predicted leaflet displacement compared to isotropy is in line with the observations made in [54, 28].

#### 3.2.3. Localized Fiber Reorientation

**Figure 8:**
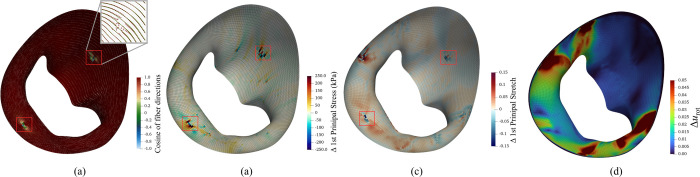
(a) Mitral valve with some fibers in the anterior and posterior belly reoriented with a zoomed-in view of the fibers before (green) and after localized fiber reorientation (red). The color shows the cosine between fibers before and after reorientation. (b) The difference in the first principal value of Cauchy stress at maximum applied pressure. The color bar is capped at ±250 kPa to improve contrast. (c) Corresponding differences in first principal stretches with color bar capped at ±0.15. (d) The relative differences in the magnitude of the displacement between references and the locally reoriented model, capped at 5% deviation. The location of localized reorientation and its effect on the stress and stretch are marked by boxes in (a), (b), and (c). We don’t observe any such significant differences in the displacement field in (d) at the location of reorientation. The fields in (b), (c), and (d) correspond to a maximum applied pressure of 10 kPa.

In general, we find the heart valve deformation to be highly resilient to fiber reorientation. For the forthcoming discussion, we consider the fiber orientation in Figure 6a to be the reference case. We reorient a few fibers in the anterior and posterior regions by defining constraints to Poisson’s equation, specifically in the anterior and posterior belly areas. The resulting fiber orientation and the cosine of the fibers in the circumferential direction with the reference case are shown in Figure 8a. A widespread dispersion of this sort is a signature of Barlow’s syndrome [55], thus the case considered here is perhaps representative of the early onset of the disease.

The difference in the first principal stress between the two cases is shown in Figure 8b. We observe significant but localized differences in the stress distribution due to this local reorientation, along with concomitant differences in the principal stretches (approximately ±15%, see Figure 8c). However, the overall valve displacement and closure quality are not noticeably affected by this local reorientation. Notably, we find $\text{Δ}u_{\text{rot}}=\frac{\left\|{\vec{u}}_{\text{ref}}-{\vec{u}}_{\text{rot}}\right\|}{\left\|{\vec{u}}_{\text{ref}}\right\|}\approx 0.05$ at low applied pressure that becomes virtually zero at maximum applied pressure in areas of localized reorientation as shown in Figure 8d. Clearly, the origin of this stress difference is due to differences in the strain field originating from localized heterogeneity in a strain-stiffening material that will be masked by the large deformation experienced by the leaflet in common medical imaging procedures.

#### 3.2.4. Global fiber reorientation and chord degradation

**Figure 9:**
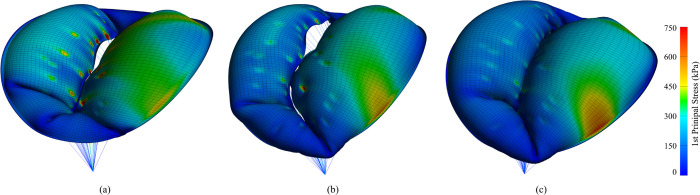
Deformed state of the heart value at maximum applied pressure with (a) global fiber reorientation, (b) reoriented fiber and compliant chord, and (c) with compliant chord but no fiber reorientation. The color bar shown in (c) applies to all panels and represents the first principal stress.

At the end of the spectrum, we investigate the effect of global reorientation of the fibers by rotating the local material axes by 90 degrees about the normal direction (i.e., value of material parameter a and b swapped) for the transversely isotropic case. This kind of flipped behavior is observed experimentally with possible links to calcium deposition [10, 56]. We find significant differences in the heart valve deformation due to this global reorientation. Specifically, we observe valve regurgitation in this case, which is clinically relevant. This deformed configuration is shown in Figure 9a.

Along with the leaflet material, the selection of the geometry and material model for the chord can significantly affect the deformation of the valve [57]. In particular, we find that a global reorientation of fibers with more compliant chords leads to a more severe valve regurgitation and billowing of the leaflet. This is shown in Figure 9b, where the chord is made compliant by reducing the exponent in the Ogden model ( $m_{k}=15$ to 5 in Eq. 10) with other parameters held constant. Without a global fiber reorientation, the valve still closes well with this compliant chord, though a significant billowing in the leaflet is observed. This result is presented in Figure 9c. Therefore, the material selection for leaflet and chord, as well as the fiber orientation, are all important factors that affect the deformation of the valve.

## 4. Discussion

The anisotropic hyperelastic model presented in this work is suitable for modeling novel behaviors of soft fibrous materials, as evident from the preceding results. A case study involving mitral valve deformation further supports this claim. Additionally, analysis reveals important insights into mechanical deformation of heart valve and risk factors associated with fiber orientation driven anisotropy and heterogeneity. The highlights of this work and their implications are briefly discussed in this section.

### 4.1. Feasibility of Proposed Constitutive Model

The primary objective and contribution of this work is the proposed octahedral fibrous material model that captures various unique properties of soft tissues. In particular, we demonstrated that exponential stiffening observed in soft biological tissue is well approximated by the material model with tunability built in through the exponents in the energy function. Similarly, large Poisson’s contraction is captured effectively by controlling the ratio of the exponents and stiffness of the fibers. The reverse Poynting effect observed in the material is captured by the particular arrangement of fibers in a cuboid unit cell. Finally, the fiber model considered here can replicate the compression behavior of dry network material with (optional) ground matrix material that represents bio-polymers, fluid, and other organic matter. Overall, the material model is ideal for fibrous material with orthotropic, transverse isotropic, and isotropic symmetry that is found in various biological tissues. In contrast to conventional anisotropic formulations that often prescribe fiber families or use orientation distributions to represent fiber dispersion, the present model defines the local fiber architecture through the vectors $\vec{a}$, $\vec{b}$, $\vec{c}$. These vectors can be informed by imaging-derived fiber orientation data and provide a path toward patient-specific modeling when patient-specific imaging data are available.

### 4.2. Effect of Fiber Orientation on Mitral Valve

**Figure 10:**
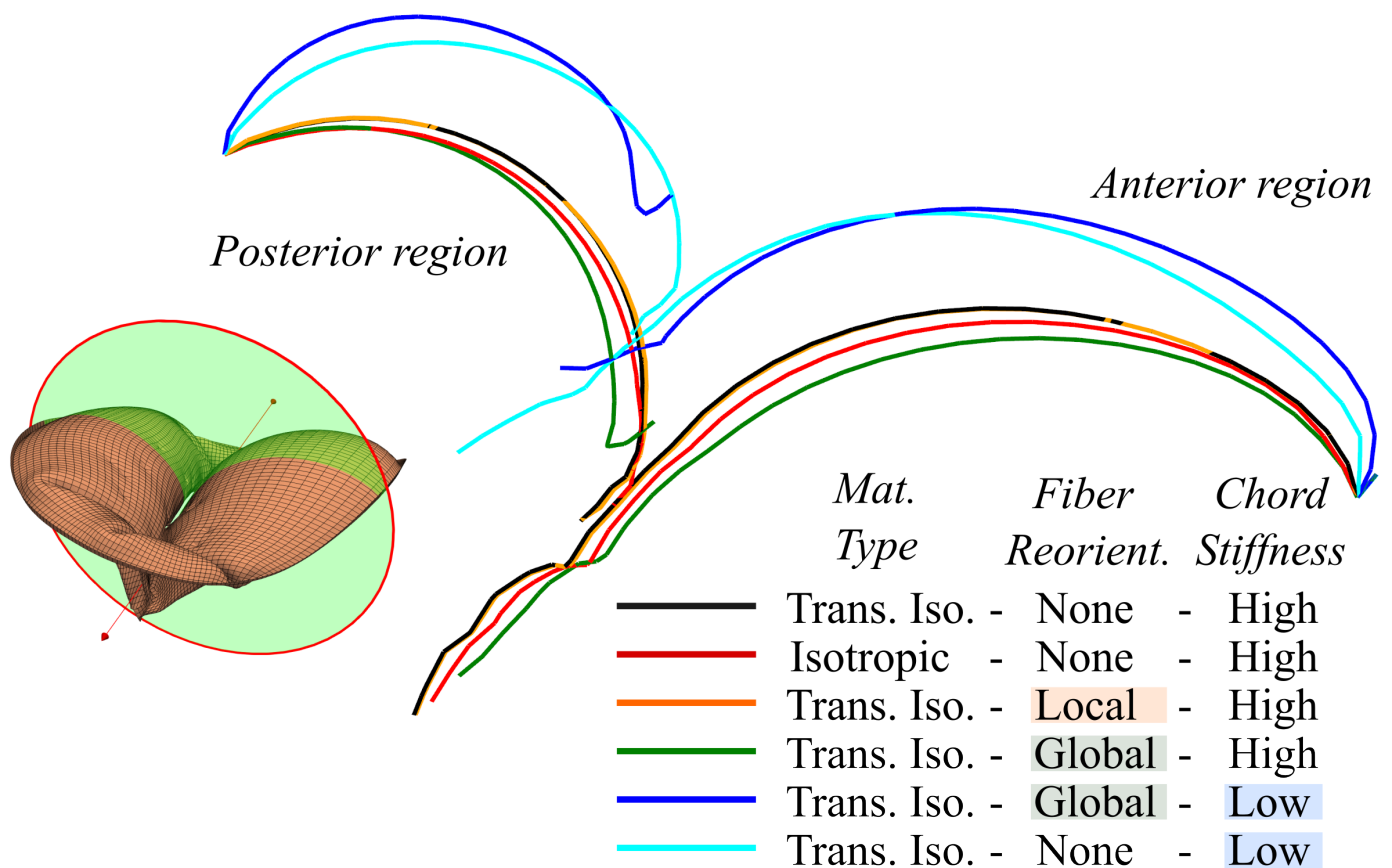
Sliced view of the heart valve at maximum applied pressure for various material model types (transverse isotropic and isotropic), fiber reorientation (Reference/None, Localized, and Global), and chord stiffness (High and Low) considered.

The mitral heart valve case study presented in Section 3.2 suggests that the isotropy assumption has a negligible effect on the mid-systolic leaflet geometry and, by extension, the predicted immediate functional outcome. However, this finding should not be interpreted to mean that fiber orientation is mechanically unimportant. Instead, our results show that fiber orientation can substantially influence the local stress and strain fields (Figure 8), which are not captured by isotropic formulations and may have important implications for cardiovascular tissue remodeling and disease progression. Localized fiber reorientation, for example, leads to a perturbation in the stress field that may not be otherwise noticeable in the displacement field. A global fiber reorientation with or without chordal degradation will lead to clinically significant outcomes. Finally, the model and specification of the chordal material can strongly regulate the effects of the fiber orientation driven anisotropy and heterogeneity. These observations are summarized in Figure 10, which shows the sliced view of the deformed heart valve for the various material and chord properties considered in this work and highlights the need for further investigation into the effect of fiber orientation-induced anisotropy.

In closing, we draw attention to the case of localized fiber reorientation. Two valves with comparable geometry but with different local fiber orientation may have similar deformation and closure quality, but significantly different spatial stress distribution. This points to a potential complexity in diagnosis and risk factor in the clinical context. First, medical imaging generally focuses on the displacement field of the heart valve, so this will remain undiagnosed. Second, individual differences may make it difficult to isolate localized fiber reorientation even if fiber orientation information can be extracted. Note that principal stresses are associated with directions - thus direct comparison in Figure 8b is somewhat ambiguous. Regardless, the fact that the difference in stress field is correlated with the fiber reorientation suggests effect of local reorientation is not fully masked by the surrounding. Therefore, this is an important effect of fiber reorientation that demands further studies to assess the generalizability of the observation, the role of material/model selection, and its long-term effects. Finally, since the altered stress state is linked to an altered strain state that can be approximated readily [58], we recommend incorporating strain information in clinical decision making whenever possible.

### 4.3. Limitations

We discuss the limitations of this work in two segments: first, related to the material model, and then related to the case study. The primary limitation of the material model is the complex energy function definition arising from a relatively large number of parameters and strong non-linearity. This limited our ability to provide an analytical solution for boundary value problems that are not only useful for material parameter calibration but also shed light on the role of various deformation mechanisms.

We mitigated difficulty in calibration to a great extent with the framework described in Section 2.2. However, our material model struggles to capture the initial toe region of the experimental data. This is a consequence of the fact that an exponential-like function is being approximated with a polynomial, and the loss function is overtly influenced by the very large deformation regime of the data. This is a common issue with many material models and optimizer combinations, and we are currently exploring alternative optimization approaches, e.g., including adaptive weights, to address this issue. We also note that the calibrated parameters are not unique. We expect that additional experimental data, e.g., from uniaxial tension tests, can help constrain the optimization problem and lead to a better solution.

In analyzing the deformation of the mitral valve, we made simplifying assumptions regarding the geometry and applicable boundary constraints to facilitate efficient numerical simulations, though it remains competitive with the FE models typically used in literature. However, due to numerical nature of the results, the observations made here need further assessment. In particular, we draw attention to the case where global fiber reorientation leads to regurgitation. Based on the influence of the chords reported in literature and observed here, a different chordal model or material parameter for the chords may nullify the regurgitation. Still, we believe these do not compromise the applicability of the proposed material model in general use, and the observations made in the case study only warrant further investigations in this direction.

## 5. Conclusion

In this work, we developed an anisotropic hyperelastic material with orthotropic symmetry to model soft fibrous materials. The material model contains 9 free parameters that can be calibrated to capture the novel mechanical behavior of various soft tissues, such as strain stiffening and reverse Poynting effect. Although the material model suffers from drawbacks due to the complexity originating from the strain energy function definition, we provide guidance toward efficient material parameter calibration and demonstrate the feasibility of this model in modeling the constitutive behavior of soft tissue with a case study focusing on the deformation of the mitral heart valve. In the process, we identify risk factors originating from fiber orientationrelated anisotropy and heterogeneity in the valve. The material model is equally applicable to general use cases such as modeling fiber-reinforced composites. Due to the flexibility of the proposed model, observed stability for complex boundary value problems, and its capability to capture various key behaviors of soft tissue, we expect it to be valuable to a broader community of scientists and engineers.

## Figures and Tables

**Figure B.11:**
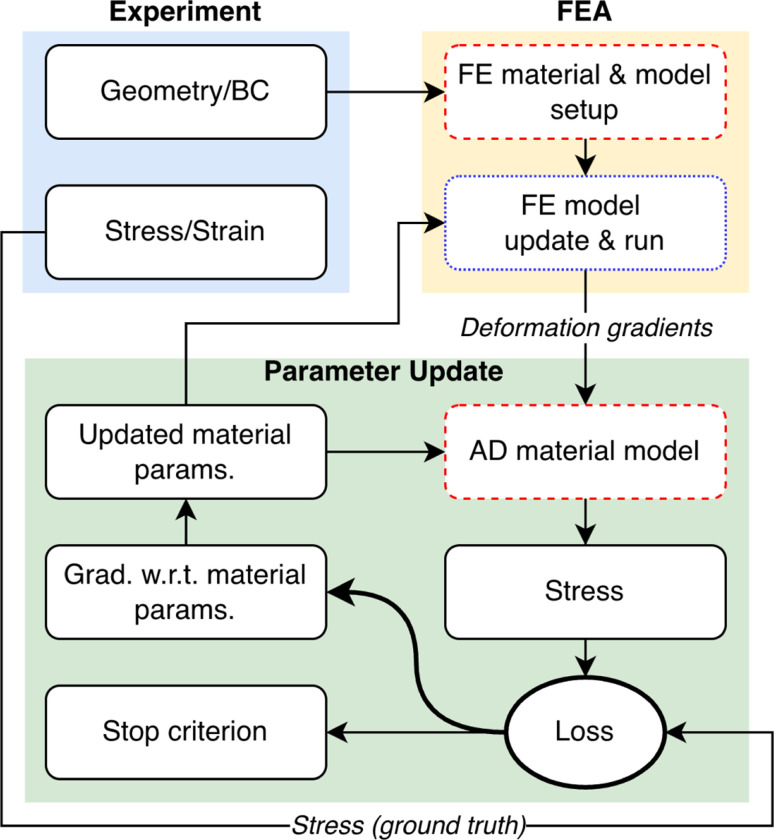
Schematic showing the material parameter calibration procedure encompassing a finite element (FE) solver and automatic-differentiation (AD) framework.

## Data Availability

Relevant data and computer code will be available at Octahedral Fibrous Model.

## Appendix A. Contribution of Fibers to Stress and Stiffness

The second Piola-Kirchoff (PK-2) stress tensor, S, due to a deformation gradient F, is defined as:

(A.1)
$$S=2\frac{\partial \psi }{\partial C},$$

where $C=F^{T}F$ is the right Cauchy-Green deformation tensor. The stress contribution from a single fiber (e.g., a center-to-face fiber) due to F is then:

(A.2)
$$S_{f}∼2\frac{\partial {\text{Ψ}}_{t}}{\partial C}=\frac{2E_{f}L_{f}}{n_{f}\;-\;1}(\lambda_{t}^{n_{f}-1}-\frac{1}{\lambda_{t}})\frac{\partial \lambda_{t}}{\partial C}$$

The contribution to the tangent stiffness tensor in the material frame can be calculated as:

(A.3)
$$\begin{array}{l} {\mathbb{C}}_{f}∼4\frac{\partial {\text{Ψ}}_{t}}{\partial C}\\ \;=\frac{4E_{f}L_{f}}{n_{f}\;-\;1}\left[\left(\left(n_{f}-1\right)\lambda_{t}^{n_{f}-2}+\frac{1}{\lambda_{t}^{2}}\right)\frac{\partial \lambda_{t}}{\partial C}⊗\frac{\partial \lambda_{t}}{\partial C}\right.\\ \;\left.+\left(\lambda_{t}^{n_{f}-1}-\frac{1}{\lambda_{t}}\right)\frac{\partial^{2}\lambda_{t}}{\partial C\partial C}\right] \end{array}$$

The contribution from center-to-corner fibers has the same functional form. The overall stress and contribution to tangent stiffness due to $\psi_{\text{aniso}}$ is the sum of these contributions, normalized by $V_{o}$.

In the above equations, the fiber stretch, $\lambda_{t}$, in the local coordinate system and its gradient with respect to a deformation applied over the unit cell are required. Assuming a fiber is defined by a vector, $\vec{v}$, in the unit cell coordinate system, the deformed length of the fiber, ρ, due to C is $\rho^{2}={\vec{v}}^{T}C\vec{v}$. Thus, the stretch equals $\lambda_{t}=\frac{\sqrt{{\vec{v}}^{T}C\vec{v}}}{v}$, where v is the magnitude of $\vec{v}$. Note, $\lambda_{t}$ reduces to anisotropic invariant, $I_{4}$, if the v is a unit vector, which is not the case here. Finally, the required gradients and their products are provided below for completeness.

(A.4)
$$\begin{array}{ll} \frac{\partial \lambda_{t}}{\partial C} & =\frac{1}{2\lambda_{t}v^{2}}(\vec{v}⊗\vec{v})\\ \frac{\partial \lambda_{t}}{\partial C}⊗\frac{\partial \lambda_{t}}{\partial C} & =\frac{1}{4\lambda_{t}^{2}v^{4}}(\vec{v}⊗\vec{v})⊗(\vec{v}⊗\vec{v})\\ \frac{\partial^{2}\lambda_{t}}{\partial C\partial C} & =-\frac{1}{4\lambda_{t}^{3}v^{4}}(\vec{v}⊗\vec{v})⊗(\vec{v}⊗\vec{v}) \end{array}$$

With application of Eq. A.4, the expression of stress in Eq. A.2 simplifies to:

(A.5)
$$\begin{array}{ll} S_{f} & ∼\frac{E_{f}v}{\lambda_{t}v^{2}\left(n_{f}-1\right)}(\lambda_{t}^{n_{f}\;-\;1}-\frac{1}{\lambda_{t}})(\vec{v}⊗\vec{v})\\ \; & =\frac{E_{f}}{v\left(n_{f}\;-\;1\right)}(\lambda_{t}^{n_{f}-2}-\frac{1}{\lambda_{t}^{2}})(\vec{v}⊗\vec{v}) \end{array}$$

Clearly, $S_{f}\to \pm \infty$ for limiting values of $\lambda_{t}$, in line with the discussion regarding the energy function. Additionally, the contribution of the fibers to the stress tensor components is dictated by the dyad $\vec{v}⊗\vec{v}$. In particular, the center-to-face fibers contribute to the diagonal components of the overall stress tensors, and center-to-corner fibers contribute to both the diagonal and off-diagonal components. Finally, $n_{f}>2$ is desired to ensure continual stiffening of the material. We consider this to be the lower limit for the exponent $n_{f}$ and $n_{c}$.

## Appendix B. Material Parameter Calibration with FEMU

Let us consider a deformation gradient that is representative of tri-axial deformation of the unit cell:

(B.1)
$$F=\left[\begin{array}{ccc} \lambda_{1} & 0 & 0\\ 0 & \lambda_{2} & 0\\ 0 & 0 & \lambda_{3} \end{array}\right]$$

Under this deformation, the stretches in the fibers simplify to:

(B.2)
$$\lambda_{f}\equiv \lambda_{a,b,c}=\sqrt{\lambda_{1,2,3}^{2}}=\lambda_{1,2,3}\;\lambda_{c}\equiv \lambda_{p1\ldots 4}=\sqrt{\frac{a^{2}\lambda_{1}^{2}\;+\;b^{2}\lambda_{2}^{2}\;+\;c^{2}\lambda_{3}^{2}}{a^{2}\;+\;b^{2}\;+\;c^{2}}}\equiv \frac{\sqrt{a^{2}\lambda_{1}^{2}\;+\;b^{2}\lambda_{2}^{2}\;+\;c^{2}\lambda_{3}^{2}}}{r}$$

Where $\lambda_{f}$ and $\lambda_{c}$ denotes stretch in center-to-face and center-to-corner fibers, respectively. This leads to the following non-zero PK-2 stress components due to $\psi_{\text{aniso}}$ :

(B.3)
$$\begin{array}{ll} S_{11,\text{aniso}} & ∼\frac{E_{f}}{\lambda_{1}\left(n_{f}\;-\;1\right)bc}\left(\lambda_{1}^{n_{f}-1}-\frac{1}{\lambda_{1}}\right)\\ \; & +\;\frac{E_{c}}{\lambda_{c}\left(n_{c}\;-\;1\right)}\left(\lambda_{c}^{n_{c}-1}-\frac{1}{\lambda_{c}}\right)\frac{a}{rbc}\\ S_{22,\text{aniso}\;} & ∼\frac{E_{f}}{\lambda_{2}\left(n_{f}\;-\;1\right)ac}\left(\lambda_{2}^{n_{f}-1}-\frac{1}{\lambda_{2}}\right)\\ \; & +\;\frac{E_{c}}{\lambda_{c}\left(n_{c}\;-\;1\right)}\left(\lambda_{c}^{n_{c}-1}-\frac{1}{\lambda_{c}}\right)\frac{b}{rac}\\ S_{33,\text{aniso}} & ∼\frac{E_{f}}{\lambda_{3}\left(n_{f}\;-\;1\right)ab}\left(\lambda_{3}^{n_{f}-1}-\frac{1}{\lambda_{3}}\right)\\ \; & +\;\frac{E_{c}}{\lambda_{c}\left(n_{c}\;-\;1\right)}\left(\lambda_{c}^{n_{c}-1}-\frac{1}{\lambda_{c}}\right)\frac{c}{rab} \end{array}$$

The stress contribution from $\psi_{\text{iso}\;}\left(=\psi_{nh}\right)$ is available in standard text such as [40]. The resulting equations can then, in theory, be used to obtain an analytical solution for boundary value problems such as the uniaxial tension test. In practice, however, this is often not feasible. An alternative approach is to use the Finite Element Model Updating (FEMU) technique coupled with an automatic-differentiation (AD) capable framework for efficient computation of the gradients in optimization process. Specifically, the AD implementation provides gradients of the loss/error term with respect to the material parameters that are then used to iteratively refine their best estimate. This framework is summarized below:

- *Step 0*: Conduction or collection of experimental data for a boundary value problem. This optimization approach does not require any specific type of experiment as long as the model and boundary conditions can be modeled in the FEA package. We use experimental data from a biaxial test. Depending on the dataset, it may be necessary to perform interpolation on the experimental data for later stages.
- *Step 1*: The material model is implemented in (i) an FEA package and (ii) in an automatic-differentiation (AD) framework. Here we choose FEBio as the FEA [42] package and JAX [43] as the AD framework. AD implementation requires the definition of the energy function only, whereas FEA implementation requires stress and tangent definitions.
- *Step 2*: We initialize the material parameters randomly and obtain an FE solution for the boundary value problem, i.e., biaxial-test. To this end, we apply the maximum stretch used in the experiments as boundary condition. The only output of interest is the full deformation gradient for the elements. We use a model composed of a single brick element to keep the computational cost to a minimum.
- *Step 3*: The deformation gradients obtained from *Step 2* are used in the JAX implementation to compute the stress with the current best guess of material parameters. The resulting stresses are compared to experimental data to compute loss. The loss function can be tailored to include soft constraints on the material parameters, e.g., enforcing transverse isotropy. We use mean squared error loss with constraints on the material parameters and symmetry enforced through inequality-based penalties with large weights.
- *Step 4*: The gradient of loss with respect to the material parameters obtained from JAX is utilized to update the current estimates of the material parameters to complete an optimization step. Here, we use the Adam optimizer to drive the optimization process.
- *Step 5*: We repeat *Step 3* and *4* until a convergence on the loss is achieved. *Step 2* is included in the loop and executed frequently as changes in material parameters lead to a change in the deformation gradient (e.g., stretches in the transverse directions).

The overall workflow is shown schematically in Figure B.11. This framework has similar applications reported in literature, e.g., in [44]. With the rapid development of automatic differentiation capable FEA packages such as JAX-FEM [59] and Tatva [60], as well as similar features in FEBio, this particular two-package coupling may not be necessary in the future. However, at this point, these alternatives are not mature enough for the current optimization task without significant effort on the end-user side. Overall, our approach strikes a good balance between the implementation complexity and optimization efficiency.

## Appendix C. Convexity of Strain Energy Function

Clearly, the overall strain energy density Ψ is convex if the individual fiber strain energy densities ${\text{Ψ}}_{t}$ are convex. Setting $g={\vec{v}}^{T}C\vec{v}$, the stretch can be written as $\lambda_{t}=\sqrt{g}/v$. The strain energy function for the fiber then becomes-

(C.1)
$${\text{Ψ}}_{t}=\frac{E_{t}L}{n_{f}\;-\;1}\left(\frac{1}{n_{f}}{\left(\frac{\sqrt{g}}{v}\right)}^{n_{f}}-\log\frac{\sqrt{g}}{v}\right)+\;\text{Constant}\;$$

The second derivative of ${\text{Ψ}}_{t}$ with respect to g is given by (assuming v=1>0):

(C.2)
$$\frac{d^{2}{\text{Ψ}}_{t}}{dg^{2}}∼\frac{E_{t}L}{4\left(n_{f}\;-\;1\right)}\left(\left(n_{f}-2\right)g^{\frac{n_{f}}{2}-2}+\frac{1}{4}g^{-2}\right)$$

The above expression is positive for all g>0 when $n_{f}\geq 2$. Since g is convex function of C, ${\text{Ψ}}_{t}$ is convex for $n_{f}\geq 2$, ensuring the overall convexity of Ψ. In the context of the overall material model, this implies that the exponent for both center-to-face and center-to-corner must obey $n_{f}$, $n_{c}\geq 2$ to guarantee convexity.
